# Anomalous Anatomical Variations of Coeliac Trunk: A Cadaveric Study

**DOI:** 10.7759/cureus.19108

**Published:** 2021-10-28

**Authors:** Mahim Koshariya, Vidhu Khare, M. C Songra, Shikha Shukla, Aryesh Gupta

**Affiliations:** 1 Department of General Surgery, Gandhi Medical College & Hamidia Hospital, Bhopal, IND

**Keywords:** coeliac trunk, variations, panagouli classification, cadaveric study, coeliac trunk anomalies, vascular anatomy

## Abstract

Background

The celiac trunk, celiac axis or celiac artery is the first major abdominal branch of the aorta. Anatomical variations of the coeliac trunk and along with the other branches of the abdominal aorta result from changes in the ventral segmental arteries supplying the digestive tube during foetal development. Panagouli performed a systemaic review and proposed a new classification describing all celiac trunk variations through a systematic review. Knowledge of the celiac trunk anatomy and any variations is clinically relevant in esophageal, gastroduodenal, hepatic, biliary and pancreatic angiographic and surgical procedures. The purpose of this study is to report the pattern of the celiac trunk and its variations in a sample of the Indian population as per the Panagouli classification.

Methods

This was an observational study done in the period from September 2018 to October 2020 in the department of surgery of Gandhi Medical College & Hamidia Hospital, Bhopal, India. Cadaveric dissection was carried out in the department of forensic medicine and toxicology after obtaining approval from the ethical committee.

Results

We did our study in 50 cadavers to look for further anatomical variations. The most common form found was true tripus Halleri. The rest of the variations noted included false tripus Halleri. Other variations were hepatosplenic trunk with left gastric artery arising from the aorta (6%), hepatosplenic trunk with no normal left gastric artery (2%), Hepatosplenic trunk with gastromesenteric trunk (2%) and coeliacomesenteric trunk (2%).

Conclusions

The congenital anomalies of the coeliac trunk have been long recognized and are of significant clinical importance as they may surprise the surgeon during surgery. Also, the wide spectrum of anomalies present in this area can be recognized by modern radiological evaluations like multi-detector helical computed tomography, MRI and magnetic resonance cholangiopancreatography (MRCP). Having the knowledge of these anatomical variations in mind may prevent inadvertent injuries during routine and complex hepato-pancreaticobiliary procedures.

## Introduction

The celiac artery, the first major abdominal branch of the aorta, has numerous anatomical variations which result from changes in the ventral segmental arteries supplying the digestive tube during foetal development. The celiac trunk trifurcates into the common hepatic artery, left gastric artery and splenic artery [1]. This trifurcation was described by von Haller [2] and is considered the classic presentation of the celiac trunk, which is known as “tripus Halleri”. Celiac trunk (CT) and hepatic artery variations and anomalies are common and usually asymptomatic. Two forms of trifurcation have been described: a “true” tripod is considered when the common hepatic artery, left gastric artery and splenic artery have a common origin, forming a hepatogastrosplenic trunk. When either one of these arteries arises before the remaining two in the course of the celiac artery, it is called a "false" tripod.

The CT presents several anatomical variations such as the absent branches (bifurcation or incomplete CT), additional branches, common origin with the superior mesenteric artery (coeliacomesenteric trunk), common origin with the superior and inferior mesenteric artery (coeliac-bimesenteric trunk) or total absence. There are several reports and studies in the available literature describing and analysing the different forms of the CT individually or in a sample of the population, while there have been numerous efforts of classification of its ramification types like Lipshutz in 1917 [2], Adachi and Hasebe in 1928 [3], Morita in 1935 [4], Michels in 1955 [5], and Prakash et al in 2012 [6]. But each of these authors proposed a classification of their own findings, which resulted in none of their studies including all variations.

Panagouli et al [7] proposed a new classification including all the described celiac trunk variations in 2013 through a systematic review.

Knowledge of the celiac trunk anatomy is clinically relevant in esophageal, gastroduodenal, hepatic, biliary and pancreatic angiographic and surgical procedures. Recent trends in surgical procedures are more towards minimally invasive surgeries and this is raising the need and the importance to know the normal vascular anatomy and to detect any anomalies and variants before the surgical interference.

The purpose of this study is to report the pattern of the celiac trunk and its variations in a sample of the Indian population, according to the classification by Panagouli et al [7].

## Materials and methods

This observational study was conducted in the Department of Surgery, Gandhi Medical College and Hamidia Hospital during the period of September 2018 to October 2020 after approval by the ethics committee (Institutional Ethics Committee, Gandhi Medical College, Bhopal - Approval no. 36124-26/MC/IEC/2018). Fifty cadavers were observed in the study period.

Methodology

Dissection was carried out in the department of forensic medicine and toxicology after obtaining approval from the ethics committee. The cadaveric dissections were done by opening the abdomen by midline and exploring and dissecting the coeliac trunk. Celiac trunk variations, accessory vessels and site of origin were recorded. Celiac trunk patterns were noted according to the Panagouli classification (Table 1).

**Table 1:** Panagouli’s classification CT: Coeliac Trunk; LGA: Left Gastric Artery; CHA: Common Hepatic Artery; SA: Splenic Artery; AA: Abdominal Aorta; SMA: Superior Mesenteric Artery

Form	Description	Prevalence
Type I (Type I of Lipshutz’s [2], Adachi’s [3], Morita’s [4] and Michels’ [5] classification)	Trifurcation of the CT (Coeliac Trunk) into LGA (Left Gastric Artery), CHA (Common Hepatic Artery) and SA (Splenic Artery)	89.42%
form 1	True tripod – common origin of LGA, CHA and SA (Tripus Halleri)	
form 2	False tripod – division into two branches while the third branch, arises earlier along the celiac trunk.	
form 2a	the LGA is the first branch	
form 2b	the CHA is the first branch	
form 2c	the SA is the first branch	
Type II (Types II, III and IV of Lipshutz’s classification, Types II, V and VI of Adachi’s classification, Types II, III and IV of Morita’s classification and Types II, IV and V of Michel’s classification and every other form of bifurcation)	Bifurcation of the CT	7.40%
form 1	hepatosplenic trunk, LGA arising from the AA (Abdominal Aorta)	
form 2	hepatosplenic trunk, no normal LGA	
form 3	hepatosplenic trunk and gastromesenteric trunk	
form 4	splenogastric trunk, CHA arising from the AA	
form 5	splenogastric trunk, CHA arising from the SMA (Superior Mesenteric Artery)	
form 6	splenogastric trunk and hepatomesenteric trunk	
form 7	hepatogastric trunk, SA arising from the AA	
form 8	hepatogastric trunk, SA arising from the SMA	
form 9	hepatogastric trunk and splenomesenteric trunk	
Type III	Additional Branches (1–6)	1.06%
Type IV (Type IV of Adachi’s classification, and Type VI of Michel’s classification)	Celiacomesenteric trunk (common origin of the CT and the SMA artery)	0.76%
Type V	Variations in the origin of the CHA	0.52%
Type VI (Type III of Adachi’s classification, and Type III of Michel’s classification)	Hepatosplenomesenteric trunk (common origin of the CHA, SA and SMA–LGA originating independently or as a branch of the others)	0.40%
Type VII (Type V of Morita’s classification)	Absence of the celiac trunk (LGA, CHA and SA are rising independently)	0.38%
Type VIII	Splenogastromesenteric trunk (common origin of the LGA, SA and SMA–CHA originating independently or as a branch of the others)	0.05%
Type IX	Splenogastric trunk giving rise to a common inferior phrenic trunk	0.008%
Type X	Celiac-bimesenteric trunk (common origin of the CT, SMA and IMA)	<0.008%

The collected data were transformed into variables, coded, and entered in Microsoft Excel (Microsoft Corporation, Redmond, USA). Data were analysed and statistically evaluated using SPSS Statistics version 20 (IBM Corp, Armonk, USA). Quantitative data were expressed in mean±standard deviation while qualitative data were expressed in percentages. Statistical differences between the proportions were tested by the Chi-square test or Fisher’s exact test. A p-value less than 0.05 was considered statistically significant.

Inclusion and exclusion criteria

All cadavers of the age group of 13 years onwards were included in the study. Mutilated, decomposed bodies, those who had undergone hepato-pancreatico-biliary surgery, and cases with hepato-pancreatico-biliary malignancy were excluded.

## Results

**Figure 1:**
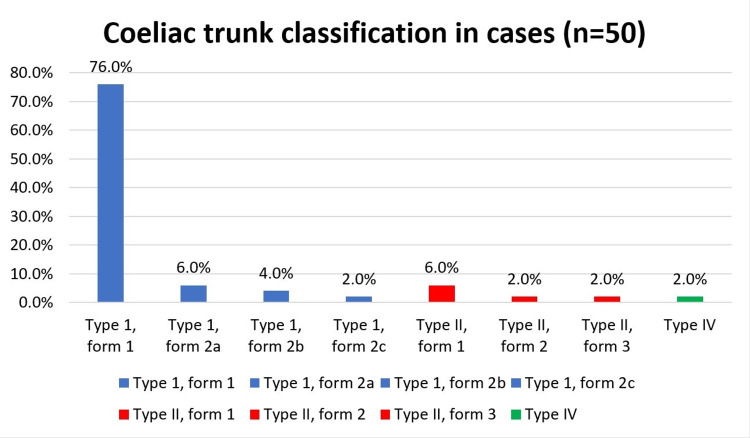
Coeliac trunk classification as per the Panagouli classification in cases in our study (n=50)

In our study of 50 cases, 28 were male and 22 were female. Most of the cases (76%) had a true tripus Halleri (Type I, form 1) (Table 2, Figure 1).

**Table 2:** Coeliac trunk classification as per Panagouli classification in cases in our study (n=50) CT: Coeliac Trunk; LGA: Left Gastric Artery; CHA: Common Hepatic Artery; SA: Splenic Artery; AA: Abdominal Aorta; SMA: Superior Mesenteric Artery

Coeliac trunk classification		Description	No.	%
Type I (n=44; 88.0%)	Type 1, form 1	True tripod (Tripus Halleri)	38	76.0
	Type 1, form 2a	False tripod The LGA is the first branch.	3	6.0
	Type 1, form 2b	False tripod The CHA is the first branch.	2	4.0
	Type 1, form 2c	False tripod The SA is the first branch.	1	2.0
Type II (n=5; 10%)	Type II, form 1	Bifurcation of the CT hepatosplenic trunk, LGA arising from the AA	3	6.0
	Type II, form 2	Bifurcation of the CT hepatosplenic trunk, no normal LGA	1	2.0
	Type II, form 3	Bifurcation of the CT hepatosplenic trunk and gastromesenteric trunk	1	2.0
Type IV		Coeliacomesenteric trunk (common origin of the CT and the SMA artery)	1	2.0

**Figure 2:**
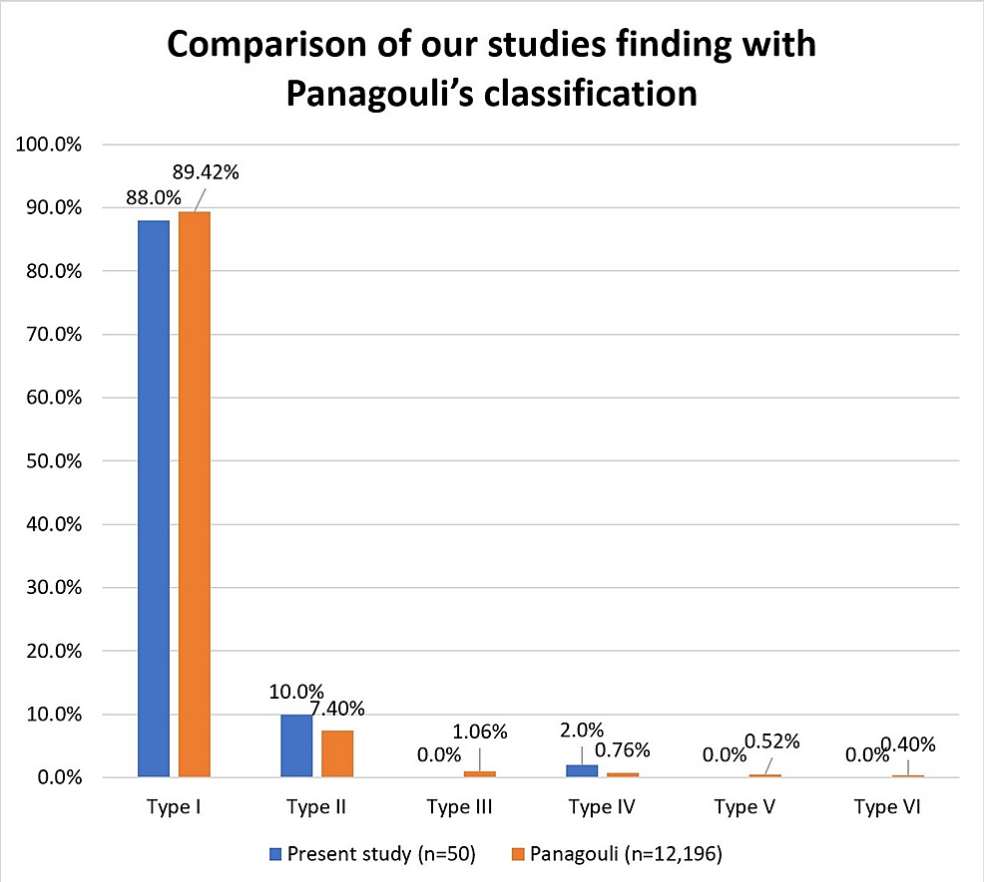
Comparison of our studies finding with Panagouli’s classification

Our findings correspond with Panagouli's classification, but they are not significant due to the large sample size used in Panagouli’s study [7] (Table 3, Figure 2).

**Table 3:** Comparison of our studies finding with Panagouli’s classification

Coeliac trunk classification (Panagouli's classification)	Present study (n=50)		Panagouli (n=12,196)		P-value
	No.	%	No.	%	
Type I	44	88.0	10906	89.42%	0.64
Type II	5	10.0	903	7.4%	0.41
Type III	0	0.0	129	1.06%	0.99
Type IV	1	2.0	93	0.76%	0.32
Type V	0	0.0	63	0.52%	0.99
Type VI	0	0.0	49	0.40%	0.99

Dissection photographs

**Figure 3:**
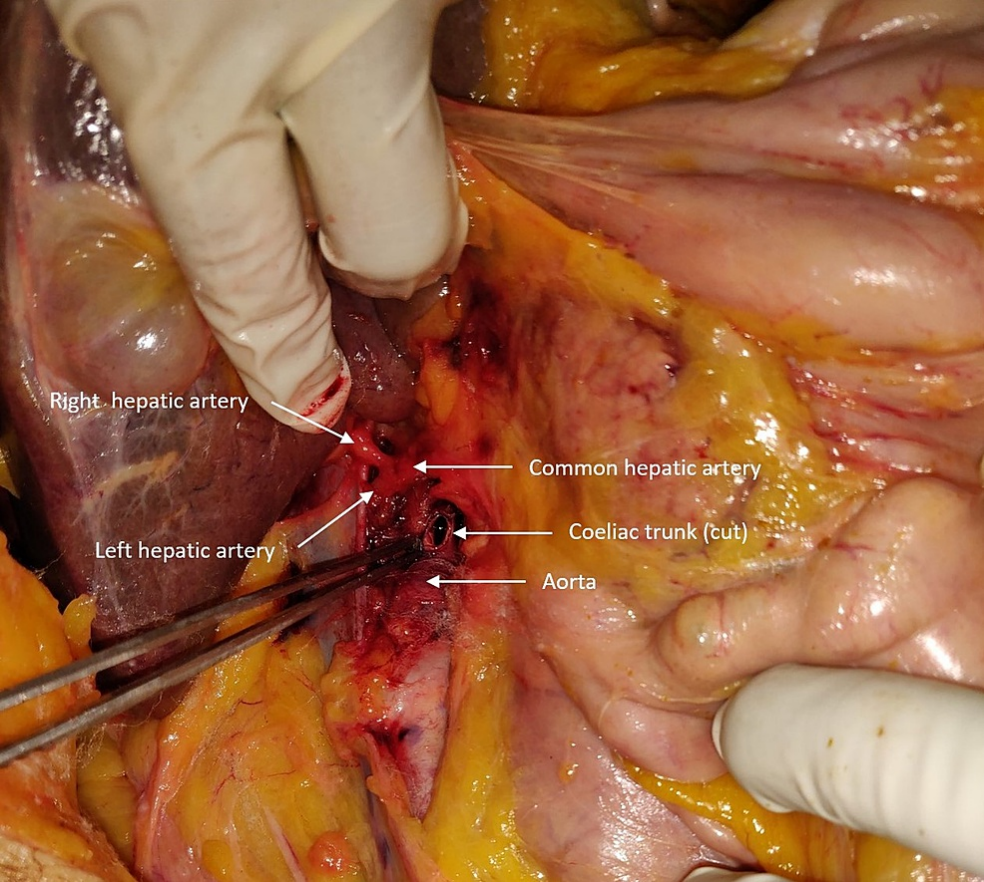
Showing the cut coeliac trunk along with visible left and right hepatic arteries in a true tripus Halleri (Type I, form 1)

Figure 3 shows the (cut) coeliac trunk and hepatic arteries visible in a case of true tripus Halleri (Type I, form 1 as per Panagouli's classification).

**Figure 4:**
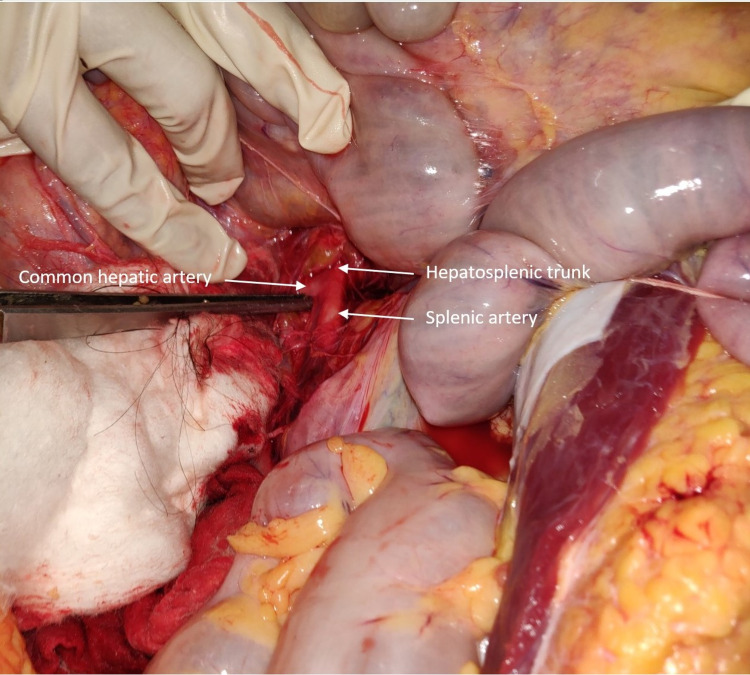
Showing a false tripus Halleri (Type I, form 2a)

Figure 4 is a dissection photograph showing a case of false tripus Halleri (Type I, form 2a as per Panagouli's classification).

## Discussion

We did our study in 50 cadavers to look for further anatomical variations. The most common form found was true tripus Halleri (Type I, Form 1 as per Panagouli's classification) (76%).

In the past, various studies have been done on abnormal anatomical variations of the coeliac trunk. We discuss here the anatomical variations we came across in our present study.

The coeliac trunk gives origin to the common hepatic artery, splenic artery, and left gastric artery simultaneously. As per the Panagouli Classification, this is termed as Type I, form 1. Lipshutz (1917) studied 83 cadavers, in which he found the true tripus Halleri configuration in 73.5% of cadavers [2]. Adachi (1928) studied 252 cadavers, in which he found this configuration in 87.7% of cadavers [3]. Prakash (2012) studied 50 cadavers, in which he found this configuration in 86% of cadavers [6]. Panagouli et al (2013) reviewed 36 studies of the past for a total sample of 12,196 cases and found this configuration in 89.42% [7]. Pinal-Garcia et al (2018) studied 140 cadavers and found this configuration in 7.1% of cadavers [8]. Juszczak et al (2020) did a cadaveric study on 50 cadavers and found this configuration in 20% of cadavers [9]. In our study, we found this configuration in 76% of cadavers.

The coeliac trunk forms a False tripus Halleri, in which one branch arises proximally before bifurcation of the coeliac trunk into the remaining two branches. Lipshutz found this configuration in 24.1% of cadavers [2], Adachi in in 8.7% of cadavers [3], Panagouli et al in 7.4% [7], Pinal-Garcia et al in 36.4% of cadavers [8], and Juszczak et al in 80% of cadavers [9]. In our study, we found the False tripus Halleri in 10% of cadavers.

These were classified further by Panagouli et al [7] based on the branch which originated proximally (Table 4).

**Table 4:** Variations of false tripus Halleri in our study

	Variation of False Tripus Halleri	Nomenclature	Prevalence in our study
1.	The Left Gastric artery as the first branch	Type I, Form 2a	6%
2.	The Common Hepatic Artery as the first branch	Type I, Form 2b	4%
3.	The Splenic Artery as the first branch	Type I, Form 2c	0%

Pinal-Garcia et al found Type I, Form 2a configuration (i.e. left gastric artery as the first branch) in 35% of cadavers [8], and Juszczak et al in 66% of cadavers [9]. In our study, we found Type I, form 2a in 6% of cadavers (Table 4). Type I, Form 2b has the common hepatic artery as the first branch. Pinal-Garcia et al found this configuration in 1.4% of cadavers [8] and Juszczak et al in 76% of cadavers [9]. In our study, we found Type I, form 2b in 4% of cadavers (Table 4). Type I, Form 2c refers to the splenic artery as the first branch. Pinal-Garcia et al found this configuration in 0% of cadavers [8] and Juszczak et al in 0% of cadavers [9]. In our study, we found Type I, form 2c in 2% of cadavers (Table 4).

There is a bifurcation of the coeliac trunk, with one artery arising from the abdominal aorta and the rest two arising as a trunk - Type II. Lipshutz found this configuration in 24.1% of cadavers [2], Adachi in 8.7% of cadavers [3], Prakash in 10% of cadavers [6], Panagouli et al in 7.4% [7], Pinal-Garcia et al in 7.1% of cadavers [8], and Juszczak et al found this configuration in 66% of cadavers [9]. In our study, we found the Type II configuration in 10% of cadavers.

The Type II configuration is further divided based on the configuration of the arteries (Table 5).

**Table 5:** Variations of Type II configurations in our study

	Variation of Type II Configuration	Nomenclature	Prevalence in our study
1.	The Left Gastric Artery arises from the abdominal aorta, followed by a hepatosplenic trunk	Type II, Form 1	6%
2.	There is a Hepatosplenic trunk, but no normal Left Gastric Artery	Type II, Form 2	2%
3.	There is a Hepatosplenic trunk and a Gastromesenteric trunk	Type II, Form 3	2%

Type II, form 1 configuration (Left Gastric Artery arises from the abdominal aorta, followed by a hepatosplenic trunk) was found in 2.8% of cadavers by Pinal Garcia et al [8], & in 16% of cadavers by Juszczak et al [9]. In our present study we found the Type II, form 1 in 6% of cadavers (Table 5). Pinal Garcia et al found Type II, form 2 configuration (i.e., there is a Hepatosplenic trunk, but no normal Left Gastric Artery) in 0% of cadavers [8], & Juszczak et al in 0% of cadavers [9]. In our present study, we found the Type II, form 2 in 2% of cadavers (Table 5). Pinal Garcia et al found Type II, form 3 configuration (there is a Hepatosplenic trunk and a Gastromesenteric trunk) in 0% of cadavers [8], & Juszczak et al in 0% of cadavers [9]. In our present study, we found the Type II, form 3 in 2% of cadavers (Table 5).

There is a common origin of coeliac trunk and superior mesenteric artery (coeliacomesenteric trunk) - Type IV as per Panagouli Classification, Lipshutz found the Type IV configuration in 2.4% of cadavers [2], Adachi in 2.4% of cadavers [3], Panagouli et al in 7.4% [7], Pinal Garcia et al in 0% of cadavers [8], & Juszczak et al in 0% of cadavers [9]. In our present study, we found Type IV in 2% of cadavers.

The rest of the variations described by Panagouli et al (2013) were not observed in our present study [7]. The absence of these variations in our study can be attributed to the fact that Panagouli et al’s sample size (12,196) was huge compared to the present study. This is also the reason that while our findings corroborate with the findings of Panagouli’s study, our findings are not statistically significant.

## Conclusions

Many variations have been established in the coeliac trunk in the context of the Indian population and an understanding of these variations is undoubtedly important for operating surgeons. Pseudoaneurysms of coeliac trunk branches may complicate several circumstances like pancreatitis, pancreatic leak following pancreatojejunostomy or pancreatogastrostomy after pancreatoduodenectomy, and leak from the pancreatic stump after distal pancreatectomy. Arterial variations in the coeliac trunk can complicate selective embolization. Complete dissection of the proper and common hepatic artery (CHA) is required during liver transplantations.

The variations of coeliac trunk are important during TACE (transarterial chemoembolisation) or radioembolisation of hepatic cancers, metastases, or pancreatic cancers. These anatomical variations are also significant in planning bariatric procedures like left gastric artery (LGA) embolization or sleeve gastrectomy.

Therefore, the congenital anomalies of the coeliac trunk have been long recognized and are of significant clinical importance as when present, may surprise the surgeon during surgery. Also, the wide spectrum of anomalies present in this area can be recognized by modern radiological evaluations like MRI, magnetic resonance cholangiopancreatography (MRCP) and multi-detector helical computed tomography. Having the knowledge of these anatomical variations in mind may prevent inadvertent injuries during routine and complex hepato-pancreaticobiliary procedures.
